# Supplementary figures and images for: Intestinal microbiome and its potential functions in bighead carp (Aristichthys nobilis) under different feeding strategies
Source: PeerJ. 2018 Dec 3;6:e6000. doi: 10.7717/peerj.6000 (PMC6283038; doi:10.7717/peerj.6000)

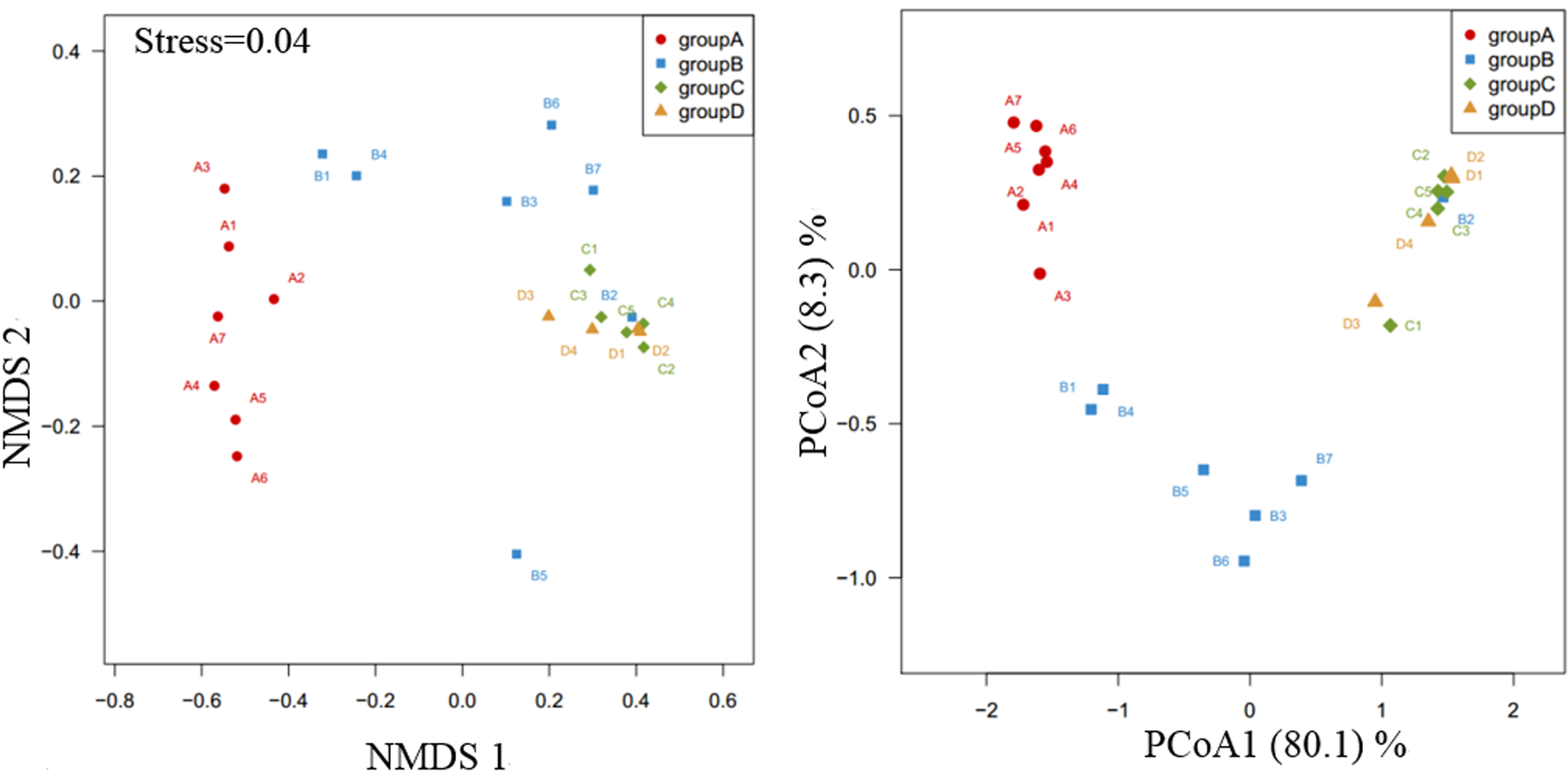

Supplement: Supplemental Information 1 — A: fertiliser; B: fertiliser+1/2 feeding; C: fertiliser+ feeding; D: feeding. [file peerj-06-6000-s001.png]

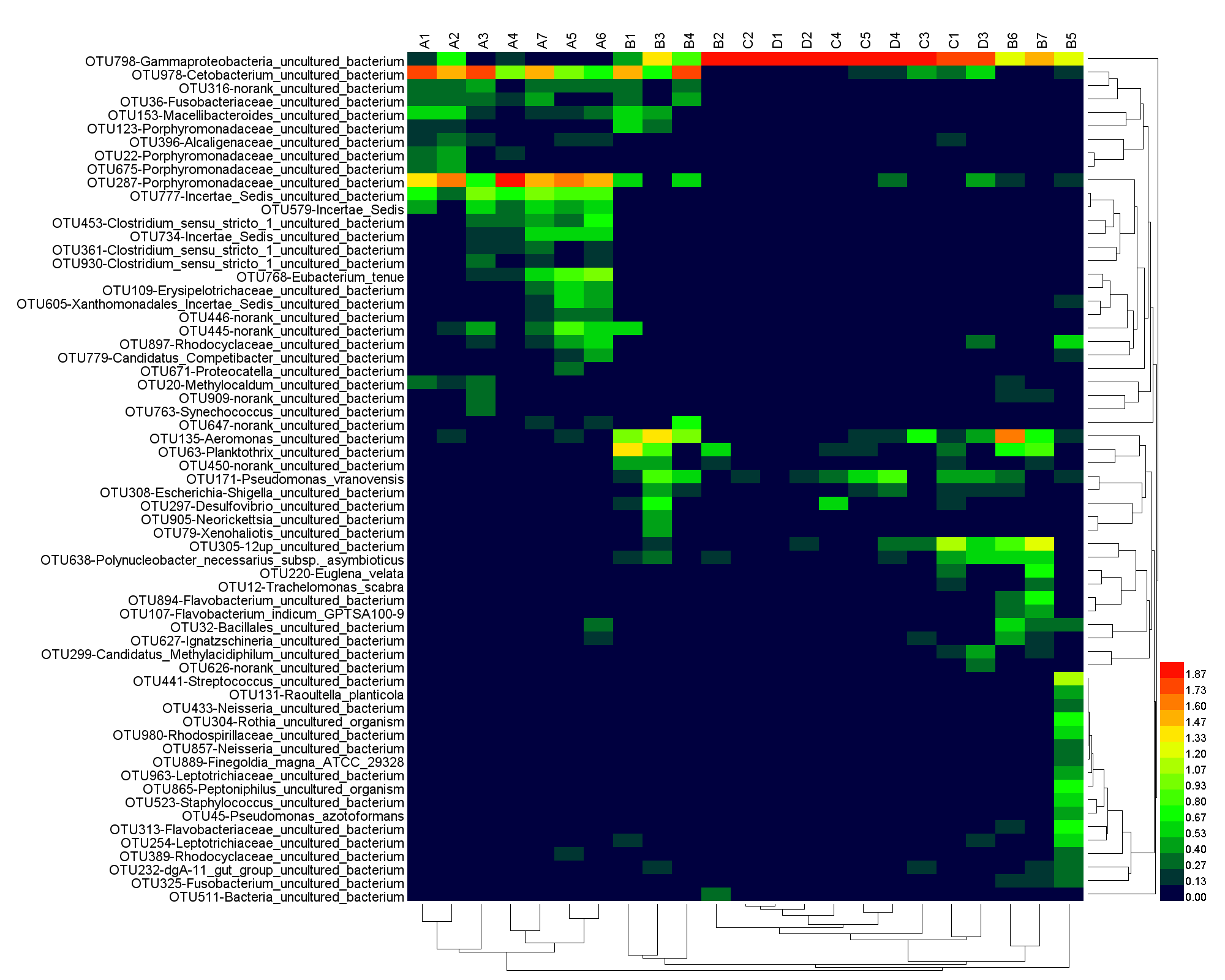

Supplement: Supplemental Information 2 — A: fertiliser; B: fertiliser + 1/2 feeding; C: fertiliser + feeding; D: feeding. [file peerj-06-6000-s002.png]

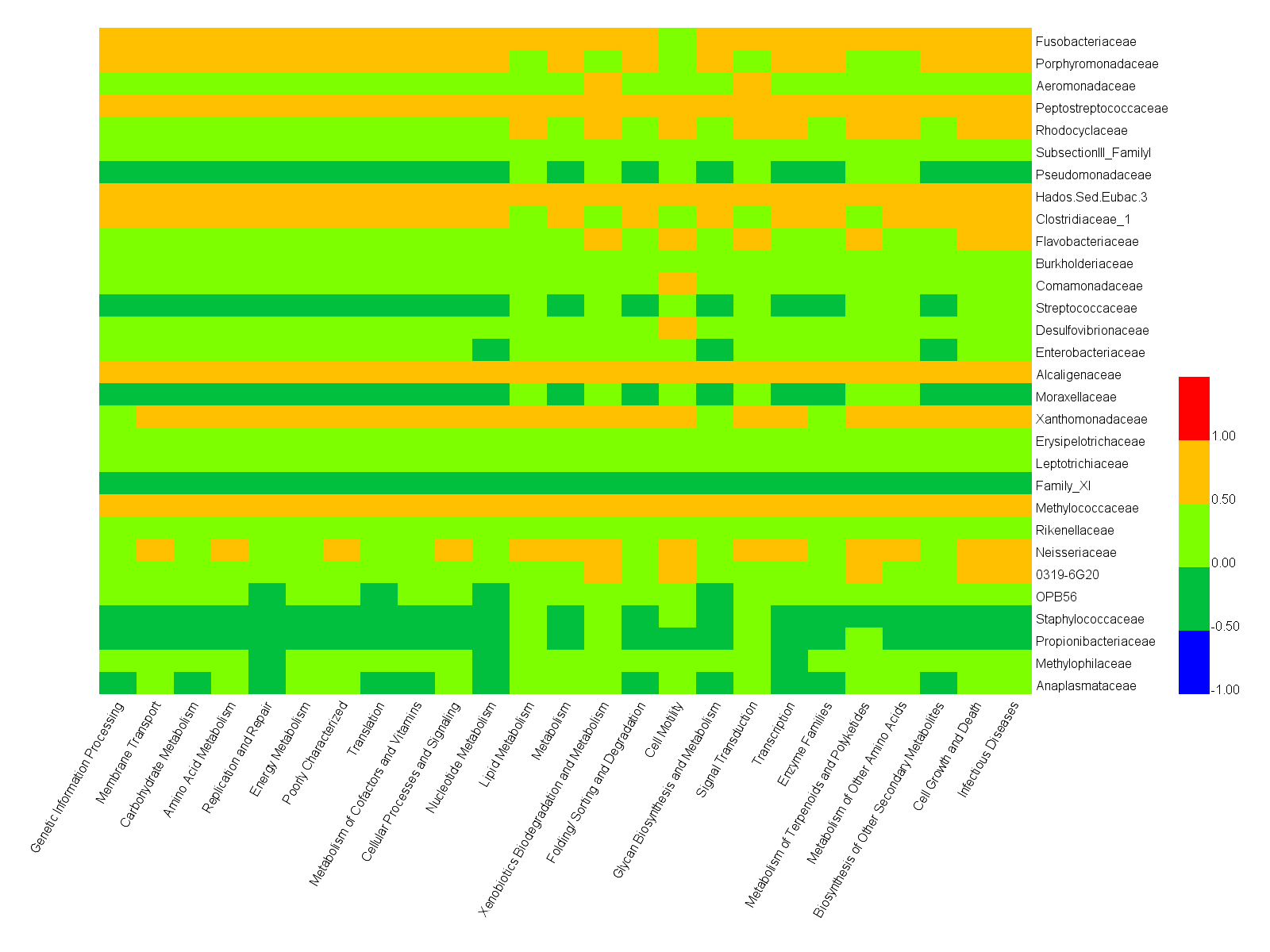

Supplement: Supplemental Information 3 [file peerj-06-6000-s003.png]
