# Supplementary material for: Intestinal microbiome and its potential functions in bighead carp (Aristichthys nobilis) under different feeding strategies
Source: PeerJ. 2018 Dec 3;6:e6000. doi: 10.7717/peerj.6000 (PMC6283038; doi:10.7717/peerj.6000)
Supplement: Supplemental Information 4 — Table S1. Main environmental factors of pond with different treatments. Mean±SD. Table S2. Relationships of environmental variables with the microbial community groups (Pearson coefficient). [file peerj-06-6000-s004.docx]

Table S1 Main environmental factors of pond with different treatments. Mean±SD

|  | Group A | Group B | Group C | Group D |
| --- | --- | --- | --- | --- |
| Water temperature T | 26.9±2.86 | 26.9±2.93 | 26.9±2.81 | 26.8±2.92 |
| Dissolved oxygen DO | 7.75±3.36 | 5.66±3.62 | 5.82±3.73 | 5.06±3.03 |
| pH | 8.29±0.38 | 8.03±0.24 | 7.88±0.31 | 7.89±0.18 |
| Transparency | 33.3±10.1 | 37.0±10.8 | 34.1±11.0 | 39.6±11.5 |

Table S2 Relationships of environmental variables with the microbial community groups (Pearson coefficient)

|  | Group A *P* value | Group B *P* value | Group C *P* value | Group D *P* value |
| --- | --- | --- | --- | --- |
| Water temperature T | -0.057 0.777 | 0.095 0.637 | 0.081 0.687 | 0.105 0.603 |
| Dissolved oxygen DO | -0.189 0.345 | -0.023 0.910 | -0.04 0.845 | 0.020 0.920 |
| pH | -0.337 0.085 | 0.122 0.545 | 0.122 0.546 | 0.170 0.396 |
| Transparency | 0.121 0.574 | -0.139 0.489 | -0.029 0.646 | -0.007 0.973 |
